# Diagnostic yield and therapeutic impact of open lung biopsy in the critically ill patient

**DOI:** 10.1371/journal.pone.0196795

**Published:** 2018-05-25

**Authors:** Carole Philipponnet, Lucie Cassagnes, Bruno Pereira, Jean-Louis Kemeny, Mojgan Devouassoux-Shisheboran, Alexandre Lautrette, Claude Guerin, Bertrand Souweine

**Affiliations:** 1 Service de Réanimation Médicale, CHU de Clermont-Ferrand, Clermont-Ferrand, France; 2 Service de Radiologie, CHU de Clermont-Ferrand, CNRS, Université Clermont Auvergne, Clermont-Ferrand, France; 3 Centre d’investigation clinique, CHU de Clermont-Ferrand, INSERM, Clermont-Ferrand, France; 4 Laboratoire d’Anatomopathologie, CHU de Clermont-Ferrand, Université Clermont Auvergne, Clermont-Ferrand, France; 5 Laboratoire d’Anatomie Pathologique, Hôpital de la Croix Rousse, Hospices Civils de Lyon, Université de Lyon, Lyon, France; 6 Service de Réanimation Médicale, Hôpital de la Croix Rousse, Hospices Civils de Lyon, Université de Lyon, Lyon, France; Hospital for Sick Children, CANADA

## Abstract

**Background:**

Open lung biopsy (OLB) is a rare procedure in intensive care units (ICUs) for therapeutic management of acute respiratory failure (ARF). The purpose of this study was to analyze the diagnostic yield, therapeutic contribution and complications of OLB in ICU patients with ARF of unclear etiology, including acute respiratory distress syndrome (ARDS) and ARDS mimics.

**Methods:**

Retrospective study conducted in a 10-bed ICU over a 13-year period. Patients undergoing OLB for ARF with undiagnosed infiltrates on CT scan were included. ARDS was defined according to Berlin criteria, and ARDS mimics as a condition looking like ARDS except for the presence of a known cause. OLB was contributive when the OLB findings yielded a specific diagnosis resulting in a change in the patients’ treatment or management.

**Results:**

Forty six patients were included (sex ratio = 2.5, median and [interquartile range] age = 69 [59–77] years, and admission SAPS II = 42 [33–50]. ARF corresponded to ARDS in 22 patients and to ARDS mimics in 16. OLB yielded 61 diagnoses in 45 patients including diffuse alveolar damage (N = 21), lung fibrosis (N = 18), and organizing pneumonia (N = 11). OLB was contributive in 37 patients (80%), including 13/16 ARDS mimickers. The main contributions of OLB were the introduction or maintenance of steroids (N = 32) and discontinuation of antibiotics (N = 9). In 4 patients OLB resulted directly in the decision to forgo life-sustaining treatment. OLB complications occurred in 16 patients (35%), in one case associated with fatal outcome.

**Conclusion:**

OLB can play a useful role in the management of ICU patients with ARF of undetermined origin, including ARDS mimickers. Further studies should be done to identify the groups of ICU patients likely to benefit from the procedure with minimum risk.

## Introduction

Definite diagnosis and optimal treatment of lung infiltrates of unclear etiologies are a major challenge in intensive care unit (ICU) patients requiring mechanical ventilation. The causes of lung infiltrates are numerous and include cardiogenic pulmonary edema, infection, alveolar hemorrhage, bronchiolitis obliterans, organizing pneumonia, inflammatory disease, fibrosis, drug reaction, cancer and hematological malignancy. In most patients a reliable diagnosis can be obtained by an extensive process comprising a complete history, physical examination, chest computed tomography (CT), bronchoscopy and microbiological analyses (cultures, serology-based and polymerase chain reaction–based detection of microorganisms in the blood or bronchoalveolar lavage (BAL) specimens). When these measures fail to identify the cause of lung infiltrates, many physicians opt for a pragmatic therapeutic approach and administer broad-spectrum antimicrobials with or without other treatments such as diuretics, and anti-inflammatory and immunosuppressive agents. However, such empirical strategies may be ineffective, unnecessary and potentially harmful. In some patients there is an obvious need to clarify the diagnosis of lung infiltrate, particularly in those who do not improve after initial evaluation or empirical therapy. In these patients, histologic examination may be useful. There are no established guidelines for the management of these patients. In general populations pulmonary samples are currently obtained by transthoracic pulmonary biopsy and transbronchial biopsy, but these procedures have been rarely reported in the ICU setting. In most studies on lung histology examination in critically ill patients, pulmonary samples were obtained by open lung biopsy (OLB), but OLB is rarely performed because of potential severe complications.

Most available data on OLB in critically ill patients derive from cases of acute respiratory distress syndrome (ARDS) reported in single center studies involving a small number of selected patients [1–7]. The findings are difficult to compare because of differences in the case mix population, definition of ARDS, indication for OLB and type of procedure. A recent meta-analysis of 14 case series involving 512 mechanically ventilated patients undergoing OLB suggests that OLB could be performed with a high diagnosis yield and an acceptable level of safety [8]. In this meta-analysis, the vast majority of patients had ARDS as defined by the American–European Consensus Criteria, but results of OLB in patients with ARF classified as ARDS and non-ARDS according to the new Berlin criteria are rare [9], and data on ARDS mimickers defined as ARDS patients with no common risk factor identified very scarce [10–11].

The purpose of our study was to report our experience on OLB in mechanically ventilated patients (with ARDS defined by Berlin criteria or without ARDS) with pulmonary infiltrates of unclear etiology after failure of the initial diagnostic evaluation.

## Methods

### Patients

We reviewed the hospital charts of all mechanically ventilated patients hospitalized in the 10-bed medical intensive care unit (ICU) of our institution between 1 January 2000 and 31 January 2014 who underwent a surgical OLB while alive for the diagnostic evaluation of pulmonary infiltrates of unclear etiology after failure of the initial diagnostic evaluation. Patients with mechanical ventilation started after OLB were excluded, as were those who underwent OLB post-mortem.

Potential cases were identified by electronic search of the database of the pathology unit. Patients were included if they met the following criteria: receiving mechanical ventilation on the day of OLB, hypoxemia (PO2/FiO2 ratio, <300 mm Hg), undiagnosed radiological pulmonary infiltrate on CT scan not related to a definite diagnosis of low respiratory tract infection, and no evidence of heart failure on echocardiography. Patients who met the Berlin criteria for ARDS at any time between ICU admission and OLB, and those who could not be classified by Berlin criteria were included (9). OLB was carried out by trained thoracic surgeons in an operating room or at the bedside in the ICU. The OLB procedure and processing are given in S1 File. Each tissue specimen was assessed for microbiological analysis and examined by two pathologists (JLK, MDS), blinded to the medical charts. In cases of discrepancies between their results, the final diagnosis was determined at a consensus meeting between the two pathologists. The readings were not compared with the original results. The data collected were recorded in S2 File.

The study was approved by our institutional review board (Comité de protection des personnes Sud-Est 6 –IRB00008526 number 2017/ CE10).

### Definitions

ARDS was defined according to Berlin criteria [9].

ARDS mimickers were defined as patients meeting the Berlin definition of ARDS without exposure to one or more common risk factors [10–11].

Definite diagnosis of low respiratory tract infection prior to OLB was defined on the basis of persistent pulmonary infiltrates on chest radiographs combined with purulent tracheal secretions and/or body temperature greater than or equal to 38.5°C or less than or equal to 36.5°C and/or peripheral blood leukocyte count greater than or equal to 10 × 10^9^/L or less than or equal to 4 × 10^9^/L and that required microbiological confirmation by quantitative culture from a BAL fluid specimen (> 10^4^ CFU/mL).

Bacterial pneumonia was defined by a positive microbiological culture of a lung sample with histopathology findings compatible with pneumonia. Cytomegalovirus and Epstein Barr virus pneumonia were diagnosed by the identification of nuclear cytopathic effect and a specific determination of the viral antigen: immunohistochemistry with antibodies to cytomegalovirus antigen and hybridization in situ with Epstein-Barr-encoded RNA (EBER) probe, respectively.

Ventilator-associated pneumonia was defined as pneumonia occurring more than 48 hours after patients have been intubated and received mechanical ventilation.

OLB was contributive when OLB findings resulted in a change in therapy or management including the decision to forgo life-sustaining treatment (DFLST) or when a specific treatment was continued on the basis of biopsy data.

Post-OLB complications were defined as deterioration of oxygenation (decrease in Pao2/Fio2 ratio >30 mm Hg), prolonged air leak, hemothorax, and biopsy-related death. Biopsy-related deaths were all those resulting from OLB procedure and deaths occurring within the 24 hours following OLB. Biopsy-related death, air leak requiring surgery and OLB-related bleeding requiring either at least two packed red blood cell transfusion within the 48 hours after OLB or an invasive procedure (angiography, surgery) for bleeding control were recorded as severe post-OLB complications.

### Statistical analysis

Results were expressed as mean ± SD or median and interquartile range (IQR) for continuous data, and as counts and percentages for categorical data. Chi-square test or Fisher’s exact test, Wilcoxon signed-rank test and Mann–Whitney U test were used as appropriate. Analysis was performed with SAS software (SAS Institute Inc). *P* values lower than 0.05 were considered statistically significant.

## Results

### OLB findings

**Fig 1:**
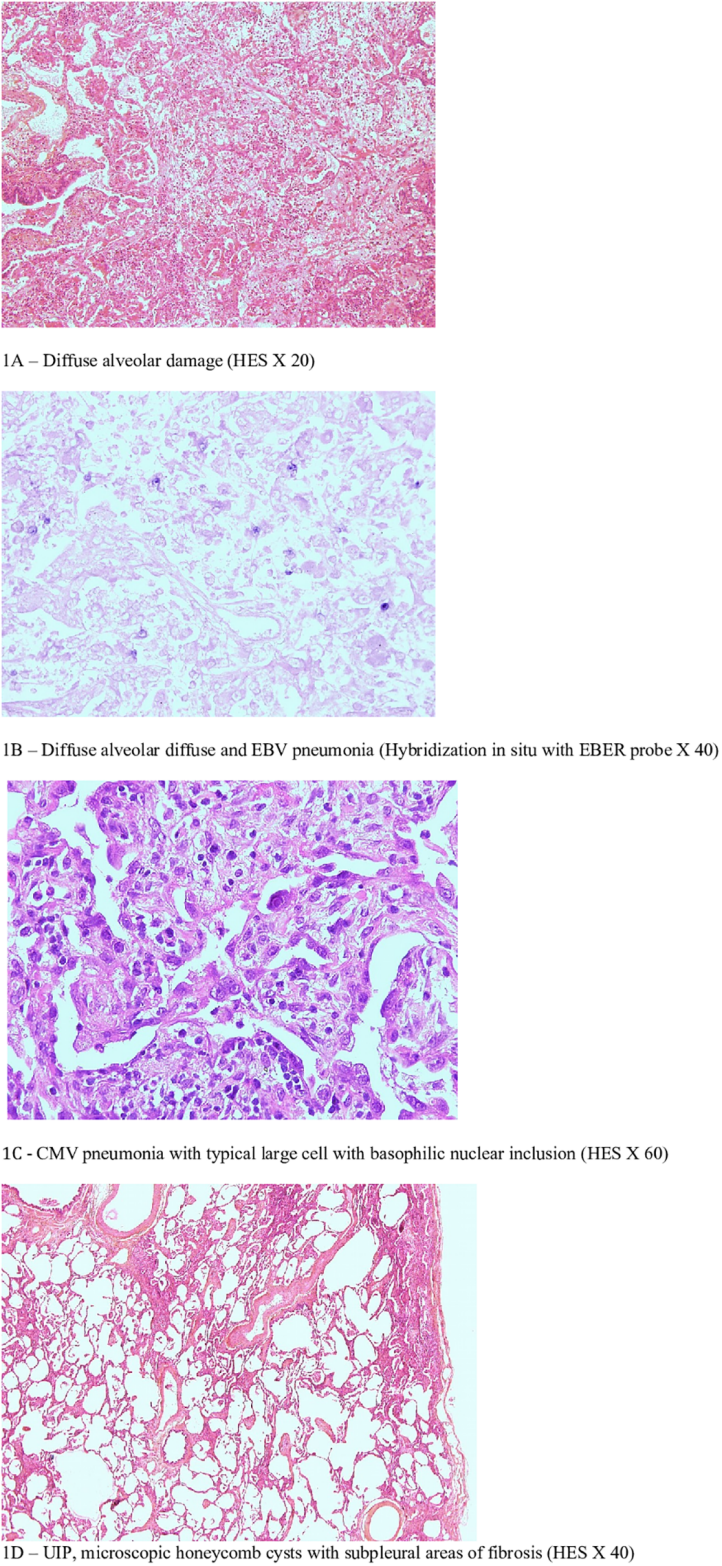
Panel of histologic patterns: DAD, UIP, and infectious pneumonia. 1A –Diffuse alveolar damage (HES X 20). 1B –Diffuse alveolar diffuse and EBV pneumonia (Hybridization in situ with EBER probe X 40). 1C - CMV pneumonia with typical large cell with basophilic nuclear inclusion (HES X 60). 1D –UIP, microscopic honeycomb cysts with subpleural areas of fibrosis (HES X 40).

The characteristics of the 46 patients of the study population are given in Table 1. Of the 46, 22 were ARDS patients, 16 ARDS mimickers and 8 non-ARDS patients. All patients had undergone BAL, CT chest scan and echocardiography prior to OLB. In 11 patients the microbiological culture of BAL performed prior to OLB identified methicillin-resistant *Staphylococcus aureus* (N = 1), *Haemophilus influenzae* (N = 1), *Stenotrophomonas maltophilia* (N = 1), *Candida albicans* (N = 3), *Aspergillus fumigatus* (N = 2), adenovirus (N = 1), Herpes simplex virus type 1 (N = 1), Epstein-Barr virus (N = 1), and cytomegalovirus (N = 2) but the criteria for defining definite low respiratory tract infection were not fulfilled. OLB yielded a pathological diagnosis in 45 patients (Fig 1). The histopathological diagnoses and subsequent outcome are given in Table 2. Diffuse alveolar damage (DAD) was observed in 21 patients. DAD was the only pathological diagnosis in 5 patients and superimposed on other pathological findings in 16: pulmonary fibrosis (N = 9), organizing pneumonia (N = 3), infectious pneumonia (N = 3), intra-alveolar hemorrhage (N = 1). Of the patients with DAD, 13 (62%) met the ARDS criteria. In the study population, 22 patients met the ARDS Berlin criteria at the time of OLB, and DAD on OLB findings was observed in 14 (64%). In patients with ARDS, the median time between ICU admission and OLB was 4 days and 5 days (P = 0.24) for those with and without DAD, respectively, and hospital mortality occurred in 9/14 and 4/8 (P = 0.66). The histopathological diagnoses and subsequent outcome of the 16 ARDS mimickers are given in S3 File.

**Table 1:** Patient characteristics in hospital survivors and non-survivors.

	All patients N = 46	Alive n = 19	Dead n = 27
Age, years^a^^,^^b^	69 [59–77]	64 [60–74]	71[60–80]
Sex ratio (male/female)	2.5	2,1	2,8
Body mass index, (kg/m^2^)^a^^,^^b^^,^^c^	25 [22–29]	25 [21–29]	25 [23–27]
Previous lung disease^a^^,^^d^	21	7	14
COPD	10	3	7
Pulmonary cancer	7	2	5
Interstitial pneumonia	5	3	2
Pulmonary hypertension	2	1	1
Asthma	1	1	0
SAPS II^a^^,^^b^	36 [33–50]	41 [30–52]	36 [33–48]
SOFA^a^^,^^b^	4 [3–8]	4 [3–8]	4 [3–7]
Reason for ICU admission^d^			
Acute respiratory failure	41	16	25
Sepsis, septic shock	3	2	1
Coma	1	1	0
Cardiac arrest	1	0	1
Days from admission to OLB^b^	4 [3–9]	4 [2–7]	4 [4–10]
SOFA^b^^,^^e^	8 [4–11]	7 [4–8]	11 [4–13]
Bilateral infiltrate on CT scan^d^	42	17	25
ARDS^d^	22	8	14
OLB in operating room^d^	43	19	24
Bedside OLB^d^	3	0	3
Systemic corticosteroids^d^^,^^e^	12	6	6
Systemic antibiotics^d^^,^^e^	29	11	18
Prophylactic heparin^d^^,^^e^	17	4	13
Therapeutic heparin^d^^,^^e^	12	6	6
Hemoglobin^b^^,^^e^	10 [9–11]	10 [9–11]	10 [9–11]
Platelet count < 50 G/L^d^^,^^e^	3	0	3
Platelet count 50–150 G/L^d^^,^^e^	9	3	6
Platelet count >150 G/L^d^^,^^e^	34	18	16
Prothrombin time < 3 sec^d^^,^^e^	34	17	17
Prothrombin time 3–6 sec^d^^,^^e^	12	4	8
Contributive OLB^d^	37	16	21
ICU length of stay^b^	17 [13–25]	15 [13–25]	19 [13–25]

^a^, on ICU admission.

^b^, median and interquartile range.

^c^, 4 missing data because of missing height.

^d^, number of patients.

^e^, on the day of OLB.

ARDS, acute respiratory distress syndrome; COPD, chronic obstructive pulmonary disease; CT, computed tomography; ICU, intensive care unit; OLB, open lung biopsy; SAPS II, simplified acute physiology score; SOFA, sequential organ failure assessment score.

**Table 2:** Histopathological findings in the 46 patients and subsequent outcome.

Histopathological findings		Patients^a^	ICU mortality^a^
Pulmonary fibrosis		18	13
	UIP	7	5
	Drug-induced	4	2
	Systemic disease	2	1
	Post radiotherapy	2	2
	NSIP	3	3
Organizing pneumonia		11	6
DAD only^b^		5	2
Infectious pneumonia^c^^,^^d^		4	3
Neoplasia		2	1
Desquamative interstitial pneumonia^e^		1	1
Respiratory bronchiolitis		1	0
Pulmonary alveolar proteinosis		1	0
Pulmonary infarction		1	1
Normal		1	0

^a^, Number of patients.

^b^, DAD was also observed in 16 other patients but combined with other lesions: pulmonary fibrosis (N = 9), organizing pneumonia (N = 3), infectious pneumonia (N = 3), intra-alveolar hemorrhage (N = 1).

^c^, EBV, CMV, nocardia, adenovirus.

^d^, no ventilator-associated pneumonia.

^e^, 68 years old patient with tobacco use having OLB for severe hypoxemia.

DIP was diagnosed, the patient died 49 days post OLB due to persistent hypoxemia. DAD, diffuse alveolar damage; DIP, desquamative interstitial pneumonia; ICU, intensive care unit; NSIP, Non-specific interstitial pneumonia; UIP, usual interstitial pneumonia.

OLB was contributive in 37/46 patients (80%). The contribution of OLB was not different between ARDS mimickers and ARDS patients: 13/16 (81%) vs 18/22 (82%), P = 1 (Fisher's Exact Test).

**Fig 2:**
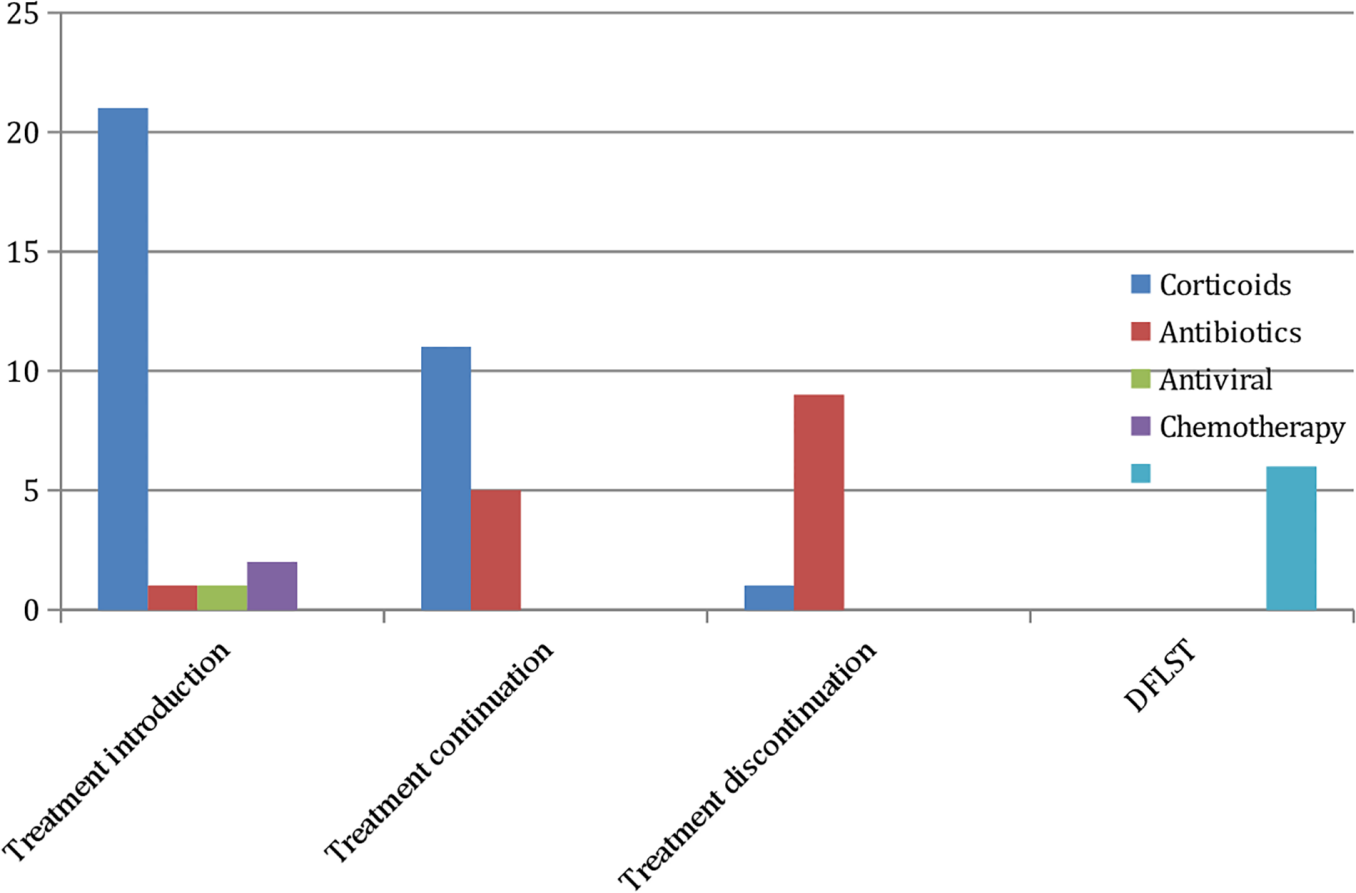
OLB contribution, treatment decisions induced by OLB results.

OLB findings yielded 57 therapeutic decisions that led to initiation of treatment in 25 cases, continuation in 16, discontinuation in 10, and DFLST in 6 (Fig 2). The DFLST was directly established from OLB findings in 4 patients because the histopathological examination showed severe fibrosis in a context of severe hypoxemia requiring mechanical ventilation. The median and IQR duration between OLB and death was 6 [4–7] days, as compared to 9 [5–17] days in the general population. DFLST was further indicated in two additional patients after failure of a specific treatment guided by OLB results, corticoids initiated for post radiation fibrosis (N = 1) and for toxic pneumonia (N = 1). The contribution of OLB in the 16 ARDS mimickers are shown in S4 File.

### OLB complications

The median and IQR values of SOFA and the respiratory-subscore on the day of OLB and on the following day remained unchanged, 8 [4–11] and 3 [2–3], respectively. There was a significant decrease in the PaO_2_/Fi0_2_ ratio on the day following OLB as compared to the values observed prior to procedure, 149+/-73 mmHg vs 180+/-89 mmHg (Wilcoxon Signed-Rank Test, P < 0.0001), respectively. The median and IQR duration of pleural chest drainage exposure after OLB in the study population was 4 [3–6] days. Post-OLB complications were observed in 16 patients, including a 36-year-old immunocompetent patient admitted to the ICU for profound hypoxemia and multi-organ failure who died a few hours after surgery. Death was not due to the OLB procedure. However, since all deaths occurring within 24 hours after OLB were a priori considered as OLB-related, this event was recorded as a complication of OLB. The 15 other patients developed 16 post-OLB complications: pneumothorax (N = 9), prolonged air leak (N = 3), bleeding requiring red blood cells transfusion (N = 2), hemothorax (N = 1), and surgical site infection (N = 1).

## Discussion

The present report shows that OLB carries a significant diagnostic and therapeutic yield in highly selected mechanically ventilated patients with ARF of unknown origin. OLB complications occurred in more than one third of the patients but the actual effect of the complications on outcome is difficult to determine owing to underlying medical conditions and the severity of the acute illness.

### Diagnostic yield of OLB

Two recent case series involving 1,205 [12] and 512 [8] ICU patients undergoing OLB, mostly for ARDS, reported the diagnoses provided by OLB. The two most common were pneumonitis / fibrosis/ interstitial lung diseases, made in 25% of cases (8,13) and infections, made in 23.5% [12] and 20% [8] of cases. In our study, these diagnoses were established in 39% and 9% of cases, respectively. The differences in the distribution of diagnoses obtained by OLB across the studies could be attributable to differences in case-mix populations, indications for OLB, duration of stay and of mechanical ventilation prior to OLB, diagnostic work-up performed before OLB, and definitions of diagnosis/classifications of histological findings. We identified only one case of bacterial pneumonia, probably because all patients underwent BAL prior to OLB and therefore those with bacterial pneumonia diagnosed on BAL culture results did not undergo OLB. Furthemore, the high rate of antibiotic patients on the day of the OLB may explain this result. As reported elsewhere [8,12], viral pneumonia was more frequently observed than bacterial pneumonia probably because the diagnosis of viral pneumonia is more difficult to obtain by a less invasive procedure. DAD, which is considered the pathological hallmark of ARDS [13], was observed in 46% of cases. We observed a -64% rate of DAD in patients who fulfilled the criteria of the Berlin definition of ARDS, in whom OLB was performed at a median time of 4 days after ICU admission. This result is in keeping with those of two recent studies on OLB involving 83 [1] and 101 ARDS patients [5] that reported DAD rates of 58% and 56%, respectively. Three of our patients with ARDS did not have DAD and OLB yielded the diagnosis of organizing pneumonia. Differentiating organizing pneumonia from the organizing phase of DAD can be difficult on a small lung sample with a reduced amount of tissue. The diagnosis of an organizing phase of DAD rather than that of organizing pneumonia was based on the presence of the following features: diffuse rather patchy lung involvement, interstitial thickening rather than purely intraluminal polypoid plugs, foci of hyaline membranes and fibrin, and vascular fibrin microthrombi [1]. We failed to observe the classical relationship between DAD and mortality in patients with ARDS [2,14] probably because the population size of our subgroup of patients with ARDS was too small. Interestingly, we observed histological features of DAD in one third of the patients without ARDS. Our study is the first to report the incidence of DAD based on OLB findings in a mixed population of patients with and without ARDS when the Berlin criteria are used to define ARDS.

### OLB contribution

Several studies have shown that OLB leads to changes in the management of patients in 49 to 92% of cases [1,3–7,15–23]. In one study, contributive OLBs were associated with an improvement in outcome [4]. In a recent meta-analysis pooling data from 14 case series, OLB was associated with a treatment alteration in 78% of patients [8]. The-80% rate of contributive OLBs observed in our study is therefore consistent with that reported in the literature. Like many authors, we found that the most frequent therapeutic change implemented after OLB was the decision to initiate, continue, or adjust the dose of steroids, or to stop administration [6,17,19–22,24,25]. OLB can identify causes treatable with steroids such as organizing pneumonia, drug reaction, diffuse alveolar hemorrhage, and hypersensitivity pneumonitis, or provide sufficient information to forgo inappropriate and potentially detrimental steroid administration [26]. In our study, steroids were introduced on the basis of OLB findings in 21 cases. Because of a low clinical suspicion of a steroid responsive process, steroids were not started prior to OLB in these patients. Isolated DAD was only observed in 3 of the 21 patients. In most remaining patients, OLB yielded a diagnosis of organizing pneumonia or fibrosis.

OLB can have a major impact on the management of antimicrobials [8,12]. The most frequent changes in antimicrobials following OLB are the introduction of antivirals [3,4] and the withdrawal of inappropriate antibiotics [6,7,23]. In our study, OLB findings had a significant effect on the anti-infectious regimen resulting in the initiation or continuation of antibiotic treatment, to address specific microorganisms identified on OLB findings in five cases, and in discontinuation of treatment in nine. Eliminating unnecessary antimicrobials may reduce drug toxicity, the emergence of resistant organisms, and costs. OLB can greatly contribute to avoiding prolonged and futile intensive care therapy, limiting the suffering of patients and families, and reducing financial costs. In our work, OLB findings were a main factor in DFLST in six patients (13%) directly leading to a rapid withdrawal of vital support in four of them. In most patients, as stated in the ATS guidelines on idiopathic pulmonary fibrosis (IPF), the results of high resolution CT scan show a specific pattern and are sufficient for the diagnosis of IPF. However, in a minority of cases in which the radiologic and histopathologic patterns are discordant, the histological UIP pattern could be essential for diagnosis [27]. In the 4 patients in our study who underwent DFLST as a result of OLB findings, the radiological criteria for UIP pattern were not fulfilled and therefore OLB was performed to identify the actual etiology and yielded the diagnosis of fibrosis. In the literature, the rates of OLB findings resulting in a decision to limit the extent of care in general ICU patients ranges between 3 and 23%: 3% [4], 11% [22], 11% [23], 23% [25]. In mechanically ventilated immunocompromised patients who subsequently died the rate was as high as 71% [7].

### ARDS mimickers

Lung histological data in ARDS mimickers are scant [9]. Two recent studies [10,11] on clinical phenotypes and outcome in this population reported a prevalence between 7.5 and 8.3% among ARDS patients. They yielded conflicting results on the impact of ARDS mimics on outcome, and reported histological findings associated with ARDS mimics in only 6 patients [11]. In our study, 16 patients fulfilled ARDS mimics criteria. Fibrosis and organizing pneumonia were the most frequent histopathological findings. The contribution of OLB was comparable between ARDS patients and ARDS mimickers.

### OLB-related complications

Complications arising from the OLB are common in critically ill patients with rates between 0 and 56% [1,3,4,7,15, 17, 19–24]. Differences in patient characteristics and definitions of complications could explain this wide range of complications across studies. In a recent meta-analysis involving only mechanically ventilated patients, OLB-related complications were observed in 147 of 512 cases (29%) and consisted mainly in air leak (>70%) [8]. In our study, complications of OLB occurred in a third of patients and consisted mainly in air leak and bleeding. One patient, who was one of the three who underwent OLB at the bedside, died as a consequence of surgery. These patients could not be transported to an operating room because they had profound hypoxemia with a PaO2 / FiO2 ratio <110 mmHg despite a high PEEP. In patients with profound hypoxemia undergoing OLB, whether the contribution and safety of OLB differ between patients undergoing OLB at the bedside or in the operating room is still unknown. Hypoxemic patients receiving mechanical ventilation with 100% FIO2 and PEEP (>10 mm Hg) could be at excessive risk of severe and prolonged OLB-related complications [12].

### Study limitations and strengths

We are aware that our study has several major limitations. First, it was retrospective, and even though indications for OLB and decisions resulting from OLB were always written on the medical charts we cannot exclude the possibility that therapeutic modifications may have been under- or overestimated. Second, it was performed in a single medical ICU and thus generalization from these findings to all other ICUs is limited. Third, OLB is an extremely rare procedure in the ICU setting. The study population was highly selected. OLB was performed in less than 0.5% of the patients admitted to the ICU requiring mechanical ventilation. OLB was performed in a highly selected heterogeneous subset of patients with acute respiratory failure by a multidisciplinary team including ICU physicians, thoracic surgeons and pulmonologists. However, there is no written policy in our ICU stipulating the indications for OLB and we cannot rule out the possibility that the more severely ill patients were excluded from the procedure or died before OLB was carried out. Fourth, the histological results could be modified by treatment initiated prior to OLB and therefore can result in a wide difference of diagnosis distribution across the literature.

Our study nevertheless has several strengths. First, the review of lung samples was double-blinded. Second, all patients underwent OLB after failure of an extended standardized diagnostic work-up comprising laboratory diagnostics, thoracic CT and bronchoscopy with BAL to obtain a definitive diagnosis. Third, our study is one of the first to describe the contribution of OLB in ARDS defined according to Berlin criteria and in ARDS mimickers.

## Conclusion

OLB is a clinically useful tool in highly selected critically ill patients on mechanical ventilation with lung infiltrate of unknown etiology and persistent acute respiratory failure despite an extensive diagnostic process. OLB entails high morbidity but provides specific etiologic diagnosis in many patients that results in major changes in their management, including the withdrawal or limitation of futile care. Further research is needed to better identify the mechanically ventilated patients likely to benefit from OLB. In the future, the development of new biomarkers and techniques to image lung injury could establish specific diagnoses and guide therapy, thereby reducing the need for OLB.

## Supporting information

S1 File OLB procedure and OLB processing. (DOCX)

S2 File Data collection. (DOCX)

S3 File Histopathological findings in the 16 ARDS mimickers patients and subsequent outcome. (DOCX)

S4 File OLB contribution, treatment decisions induced by OLB results in the 16 ARDS mimickers patients. (DOCX)

## Abbrevations

ARDS: acute respiratory distress syndrome
ARF: acute respiratory failure
BAL: bronchoalveolar lavage
DAD: diffuse alveolar damage
DFLST: decision to forgo life-sustaining treatment
ICU: intensive care unit
NSIP: non-specific interstitial pneumonia
OLB: open lung biopsy
PEEP: positive end-expiratory pressure
UIP: usual interstitial pneumonia
