# Supplementary material for: Diagnostic yield and therapeutic impact of open lung biopsy in the critically ill patient
Source: PLoS One. 2018 May 25;13(5):e0196795. doi: 10.1371/journal.pone.0196795 (PMC5969763; doi:10.1371/journal.pone.0196795)
Supplement: S1 File — (DOCX) [file pone.0196795.s001.docx]

Supplemental file 1. OLB Procedure and OLB processing

OLB was carried out by trained thoracic surgeons using anterior minithoracotomy in the operating room or at the bedside in the ICU. Bedside OLB could be indicated in patients with profound hypoxemia and without pleural symphysis or increased risk of hemorrhage. Anticoagulant therapy was stopped >12 hours prior to OLB. The patient was sedated and paralyzed and ventilated in volume assist-control mode with the same settings as those used prior to the OLB. FiO2 was increased to 1.0 during the OLB procedure. Oxygen saturation was monitored continuously by pulse oximeter. The pulmonary area for OLD was determined by computed tomography, and when pulmonary infiltrates were bilateral and homogeneous, preference was given to a left-sided approach for a resection of the tip of the lingula (Miller Ann Thor Surg 1987).

A chest tubes was inserted before closing the chest. Chest radiography was obtained in each patient after the procedure and thereafter daily until the drains were taken out. Pneumothorax, air leak, fluid output and bleeding through the drains and any local complications were recorded the first week following OLB.

The lung sample was divided into sections for microbiological cultures (bacteriology, mycology and virology) and pathology. One piece of lung retrieved was sent to the pathologic laboratory to be frozen (Supplemental file 2).

For pathologic examination, the piece of lung was fixed in 10% buffered formalin, dehydrated, embedded in a paraffin block, and cut into 4 micrometer thicknesses. Slides were stained with hematoxylin-eosin-safran. Grocott, Ziehl-Neelsen and immune immunohistochemical staining (Anti-CMV) was performed for microbiological examination. The OLB pathological samples were reviewed by two pathologists (JLK, MDS) blinded to the medical charts.
