# Supplementary material for: Diagnostic yield and therapeutic impact of open lung biopsy in the critically ill patient
Source: PLoS One. 2018 May 25;13(5):e0196795. doi: 10.1371/journal.pone.0196795 (PMC5969763; doi:10.1371/journal.pone.0196795)
Supplement: S2 File — (DOCX) [file pone.0196795.s002.docx]

Supplemental File 2. Data collection

Data extracted from the medical records and files were age, sex, underlying diseases, reason for ICU admission, Simplified Acute Physiology Score (SAPS) II on admission, Sequential Organ Failure Assessment (SOFA) score on admission and on the day of OLB, ventilator settings, bronchoalveolar lavage results and CT scan findings performed prior to OLB, time from admission to OLB, laboratory test results on the day of OLB comprising hemoglobin, platelet count and coagulation parameters, post-OLB therapeutic changes, post-OLB complications, ICU length of stay and ICU mortality.
