# Supplementary material for: Diagnostic yield and therapeutic impact of open lung biopsy in the critically ill patient
Source: PLoS One. 2018 May 25;13(5):e0196795. doi: 10.1371/journal.pone.0196795 (PMC5969763; doi:10.1371/journal.pone.0196795)

Supplemental file 4: OLB contribution, treatment decisions induced by OLB results

Number of patients


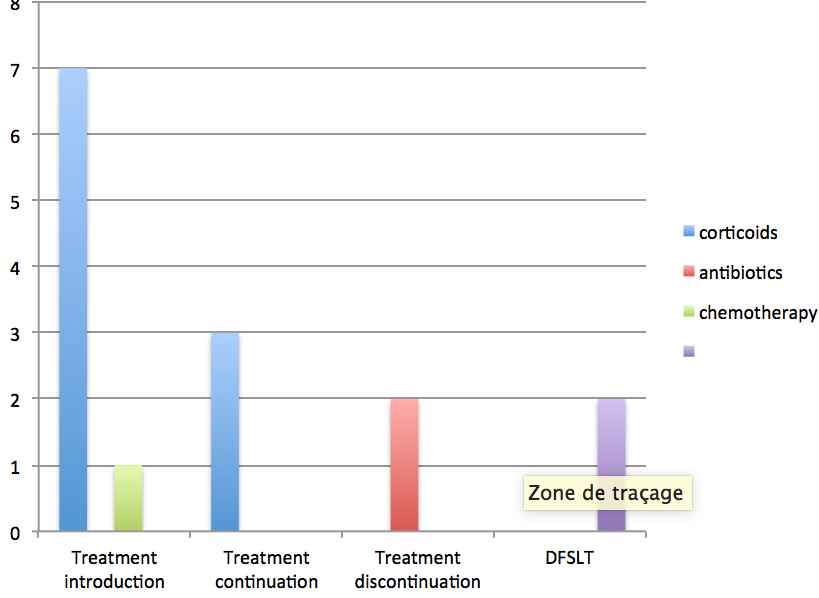

Supplement: S4 File — (DOCX) [file pone.0196795.s004.docx]
